# The Fc-mediated effector functions of a potent SARS-CoV-2 neutralizing antibody, SC31, isolated from an early convalescent COVID-19 patient, are essential for the optimal therapeutic efficacy of the antibody

**DOI:** 10.1371/journal.pone.0253487

**Published:** 2021-06-23

**Authors:** Conrad E. Z. Chan, Shirley G. K. Seah, De Hoe Chye, Shane Massey, Maricela Torres, Angeline P. C. Lim, Steven K. K. Wong, Jacklyn J. Y. Neo, Pui San Wong, Jie Hui Lim, Gary S. L. Loh, Dongling Wang, Jerome D. Boyd-Kirkup, Siyu Guan, Dipti Thakkar, Guo Hui Teo, Kiren Purushotorman, Paul E. Hutchinson, Barnaby E. Young, Jenny G. Low, Paul A. MacAry, Hannes Hentze, Venkateshan S. Prativadibhayankara, Kantharaj Ethirajulu, Jason E. Comer, Chien-Te K. Tseng, Alan D. T. Barrett, Piers J. Ingram, Trevor Brasel, Brendon John Hanson

**Affiliations:** 1 Biological Defence Programme, DSO National Laboratories, Singapore, Singapore; 2 Department of Microbiology & Immunology and Office of Regulated Nonclinical Studies, University of Texas Medical Branch, Galveston, TX, United States of America; 3 Hummingbird Bioscience, Singapore, Singapore; 4 Life Science Institute, National University of Singapore, Singapore, Singapore; 5 National Centre for Infectious Diseases, Singapore, Singapore; 6 Singapore General Hospital, Singapore, Singapore; 7 Programme in Emerging Infectious Disease, Duke-National University of Singapore Medical School, Singapore, Singapore; 8 Department of Microbiology & Immunology, Yong Loo Lin School of Medicine, National University of Singapore, Singapore, Singapore; 9 Experimental Drug Development Centre, Therapeutics Development, A*STAR Research Entities (ARES), Singapore, Singapore; 10 Department of Microbiology & Immunology and Center of Biodefense and Emerging Disease, University of Texas Medical Branch, Galveston, TX, United States of America; 11 Department of Pathology and Sealy Institute for Vaccine Sciences, University of Texas Medical Branch, Galveston, TX, United States of America; University of Alabama at Birmingham, UNITED STATES

## Abstract

Although SARS-CoV-2-neutralizing antibodies are promising therapeutics against COVID-19, little is known about their mechanism(s) of action or effective dosing windows. We report the generation and development of SC31, a potent SARS-CoV-2 neutralizing antibody, isolated from a convalescent patient. Antibody-mediated neutralization occurs via an epitope within the receptor-binding domain of the SARS-CoV-2 Spike protein. SC31 exhibited potent anti-SARS-CoV-2 activities in multiple animal models. In SARS-CoV-2 infected K18-human ACE2 transgenic mice, treatment with SC31 greatly reduced viral loads and attenuated pro-inflammatory responses linked to the severity of COVID-19. Importantly, a comparison of the efficacies of SC31 and its Fc-null LALA variant revealed that the optimal therapeutic efficacy of SC31 requires Fc-mediated effector functions that promote IFNγ-driven anti-viral immune responses, in addition to its neutralization ability. A dose-dependent efficacy of SC31 was observed down to 5mg/kg when administered before viral-induced lung inflammatory responses. In addition, antibody-dependent enhancement was not observed even when infected mice were treated with SC31 at sub-therapeutic doses. In SARS-CoV-2-infected hamsters, SC31 treatment significantly prevented weight loss, reduced viral loads, and attenuated the histopathology of the lungs. In rhesus macaques, the therapeutic potential of SC31 was evidenced through the reduction of viral loads in both upper and lower respiratory tracts to undetectable levels. Together, the results of our preclinical studies demonstrated the therapeutic efficacy of SC31 in three different models and its potential as a COVID-19 therapeutic candidate.

## Introduction

In December 2019, a cluster of human novel pneumonia cases, now named COVID-19, emerged and rapidly spread globally [1, 2]. High-throughput sequencing of patient-derived samples identified a novel beta-coronavirus, subsequently termed SARS-CoV-2, as the etiological agent. SARS-CoV-2 was found to have 79.6% sequence homology to SARS-CoV, the virus responsible for an epidemic that caused 774 fatalities during 2002–2003 [3–6]. Like SARS-CoV, SARS-CoV-2 has the potential to cause severe respiratory distress and significant mortality and morbidity [1, 2]. SARS-CoV-2 was found to bind to angiotensin-converting enzyme 2 (ACE2), the same cellular surface receptor used by SARS-CoV, via the receptor-binding domain (RBD) of the viral surface Spike protein (SP) [7].

The isolation of antibodies from the memory B cells of convalescent patients are fast becoming an attractive approach for the development of therapeutic antibodies. Such antibodies have been found to be protective against coronavirus diseases, including SARS-CoV, in animal models [8, 9]. In particular, antibodies that prevent viral SP from binding to ACE2 were highly potent at attenuating or abolishing SARS-CoV-2 infection [10, 11]. While such antibodies are not routinely used clinically to treat coronavirus infections, antibodies derived from convalescent patients have been successfully used to treat other infectious diseases, including the highly lethal Ebola virus infection with protective efficacies superior to that of small molecule antivirals [12]. This indicates the highly promising therapeutic potential of antibodies derived from convalescent patients. Not surprisingly, the B cell epitope-rich SARS-CoV-2 SP and its RBD have become the focus of numerous accelerated vaccine development programs [13–15]. However, given the current challenges associated with large-scale rollout of effective vaccines to the global population, alternative options for protective immunity, even for short periods of time, must be considered. Therapeutic and prophylactic antibodies specific to SARS-CoV-2 possess great clinical potential as they provide a much-needed option for individuals who might respond poorly to vaccination.

Multiple neutralizing antibodies against SARS-CoV-2 with therapeutic potential have been reported [16–20]. The prophylactic and therapeutic efficacies of several of these antibodies have been demonstrated in mouse, hamster and non-human primate (NHP) models. A smaller number of neutralizing antibodies have also progressed through clinical trials to be granted Emergency Use Authorization for use as COVID-19 therapeutic agents [21–23]. However, there is limited information, beyond their neutralization activities, on their mechanism(s) of action. In preclinical models, their therapeutic benefit was associated with reduced viral load and decreased lung inflammation that corresponded to lower levels of proinflammatory cytokines and chemokines (such as IL-6, CCL2 and CXCL10) which have been observed to be elevated during a COVID-19 cytokine storm [24]. Despite this, Antibody-Dependent Enhancement (ADE) of disease remains a major concern for the use of anti-SARS-CoV-2 antibodies as therapeutics. ADE can occur if Fcγ Receptor (FcγR) engagement mediates an increase in the infection of phagocytic cells or other FcγR-bearing cells that take up the opsonized viral particles [25]. Indeed, ADE has been observed in both *in vitro* and *in vivo* studies of SARS infection [26–28]. Based on observations in murine models of SARS-CoV-1, ADE might serve as a driver of dysregulated and profound inflammatory responses often associated with severe COVID-19 patients [25, 27], making the usage of specific antibodies a risk factor for enhanced diseases. While it has been reported that higher anti-SP serum IgG levels could directly correlate with the severity of disease in hospitalized COVID-19 patients [29, 30], there is currently no evidence to support an increased risk of ADE as a result of convalescent plasma administration.

Due to the potential of ADE, several ongoing SARS-CoV-2 antibody programs have chosen to use Fc isotypes that do not engage FcγR, like the IgG4 isotype, and engineered variants such as those carrying the LALA mutations [21]. However, these may be counterproductive as the signaling mechanisms underpinning the efficacies of these antibodies, particularly the ability of FcγR engagement to induce other antiviral responses, have not been evaluated.

To address these questions, we isolated and characterized a RBD-binding neutralizing IgG1 antibody, SC31, from an early convalescent patient. We showed that SC31 has potent therapeutic effects in multiple animal models. In addition, we assessed the impact of Fc functionality on its therapeutic efficacy by comparing SC31 with its FcγR-null LALA variant and demonstrated that the engagement of Fc receptors by SC31 trigger additional IFN-γ-mediated antiviral responses but do not induce ADE. We showed that both the neutralization activity and Fc-mediated antiviral responses of SC31 are required for its optimal efficacy against SARS-CoV-2 infection in the K18-hACE2 Tg mouse model for COVID-19.

## Methods

### Single cell sorting and culture

After informed consent was taken, convalescent patient samples were collected at Singapore General Hospital from patients in accordance with the approved IRB protocol (2018/3045), specifically for this study. Patient PBMCs were separated from whole blood using standard ficoll density gradients protocols then incubated at 5×10^6^ cells/ml with 10 μg/ml Twin-strep-tagged WT-spike for 1 h at 4°C in FACS buffer (1x PBS, 5 mM EDTA, 1% fetal calf serum), washed in 1xPBS and then stained with fluorescently labelled antibodies at the following concentrations (5 μl anti-HuCD19-Pacific Blue (BD Biosciences #740796), 5 μl anti-HuCD27-Alexa647 (Bio-Rad #MCA755A647), 2.5 μl-anti-HuIgG-BV711 (BD Biosciences #740796), 5 μl anti-HuCD38-PE-Cy7 (BD Biosciences # 560677), 2.5 μl anti-Strep-tagII-Alexa488 (IBA GmBH #2-1564-050) in 100 μl of FACS buffer per 10^6^ PBMC cells) for 30 min at 4°C. Cells were washed with 1xPBS and resuspended in FACS buffer containing 1:100 propidium iodide (PI) before sorting on a FACSAria Fusion. PI^-^, CD19^+^, IgG^+,^ Spike^+^ cells were sorted individually into 96-well plates containing 50 μl of IMDM culture medium, 5×10^3^ 3T3-msCD40L feeder cells [31] and supplements and cytokines at concentrations previously described without CpG and the addition of IL-21 at 10 ng/ml [32]. Cells were spun down after 8 days of culture and stored in 10 μl of lysis buffer (QuickExtract RNA Extraction Solution, Lucigen) and supernatants tested by ELISA and microneutralization.

### Antibody and viral protein expression and purification

Expression was performed using the HEK 293EBNA1-6E expression system with pTT vectors (National Research Council Canada). Antibody variable heavy and light chain sequences were cloned into pTT containing the appropriate human IgG1 heavy, kappa or lambda light chain constant regions as well as a leader sequence for secretory expression. The isotype control antibody used in these studies is specific for the influenza A H5 hemagglutinin and has been described previously [33]. For Spike protein expression, the complete ectodomain including the leader sequence (Accession No. MN908947, S gene amino acids 1–1208) together with a C-terminal Twin-strep tag (WSHPQFEK-GGGSGGGSGGS-SAWSHPQFEK), replaced the antibody sequence. Point mutations to spike protein were subsequently introduced using Quikchange site-directed mutagenesis kit (Agilent). For RBD expression, only amino acids 331–524 were cloned in together with Twin-strep tag and the leader sequence. Purified plasmid was transfected into HEK 293EBNA1-6E suspension cell culture in F17 media (Thermo Fisher Scientific) at a concentration of 1.0 mg DNA/L of culture with branched polyethylenimine (Sigma-Aldrich) at 3:1 w/w ratio of PEI:DNA. Culture supernatant was harvested after 7 days and purified by HPLC using StrepTactinXT Superflow High Capacity Column (IBA Life GmBH) or MabSelect SuRe Column (Cytiva) according to the manufacturer’s protocols for viral proteins or antibodies respectively. Clinical grade antibody was manufactured from a stable CHO cell with full analytical release testing confirming antibody purity and potency.

### Virus culture and microneutralization

SARS-CoV-2 virus obtained from a patient nasal swab was cultured in VeroE6 cells (ATCC CRL-1586) and supernatant harvested on observation of 90% CPE (hCoV-19/Singapore/3/2020). Antibodies at indicated concentrations was incubated with 100 TCID_50_ of virus and 2×10^4^ VeroE6 cells in 100 μl of culture media (MEM/2% FCS) in 96-well flat bottom plates and incubated for 72 h. The Neutralization was measured using Viral Toxglo reagent (Promega) to determine the percentage of cell survival relative to “no virus” and “virus only” controls. For the initial screening of B cell supernatant, 12.5 μl of supernatant was mixed with 25 TCID_50_ of virus instead.

### ELISA

To determine antibody affinity to RBD, WT or mutant spike protein, 2 μg/ml purified protein in binding buffer (100 mM Tris-HCl, 1 mM EDTA, 150 mM NaCl, pH 8.0) was coated onto StrepTactin XT 96-well ELISA plates (IBA GmBH) for 2 h. Plates were washed with PBS and antibody diluted into 2% BSA/PBS blocking solution at indicated concentrations was then incubated for 1h before washing thrice with PBS/0.05%Tween. For binding at different pH, antibodies were incubated in 1xPBS adjusted to the appropriate pH with hydrochloric acid with 0.5% BSA as block. Antibody binding was detected using 1:5000 anti-huIgG Fc-HRP conjugated secondary antibody (Thermo Fisher Scientific #31413) diluted in blocking solution incubated for 1 h. Plates were then washed thrice with PBS/0.05% Tween and once with PBS. After washing, plates were developed with colorimetric detection substrate 3,3′,5,5′-tetramethylbenzidine (Turbo-TMB; Pierce). The reaction was stopped with 2 M sulphuric acid, and absorbance was measured at 450 nm

### Binding inhibition assay

20 mM Twin-strep-tagged viral protein was incubated with antibody for 1 h in 50 μl FACS buffer. 5×10^4^ huACE2 expressing CHO cells (a kind gift from A/Prof Tan Yee Joo, National University of Singapore) in 50 μl FACS buffer was added and incubated for a further 1 h. Cells were spun down at 300 g for 5 min, washed twice with PBS, and then stained with 1:50 Alexa488 conjugated anti-Strep-Tag II antibody (IBA GmBH #2-1564-050) for 30 min in 50 μl FACS buffer. All incubations were carried out at 4°C. Cells were finally washed once with 1x PBS and stained with 1:100 propidium iodine (PI) and analyzed on a BD FACS Canto II. PI^-^ cells were gated and binding was measured by Alexa488 fluorescence intensity.

### Antibody Dependent Cellular Cytotoxicity (ADCC) assay

Antibody Dependent Cellular Cytotoxicity was tested using a Jurkat reporter cell line stably expressing FcγRIIIa and an NFAT response element driving downstream expression of firefly luciferase as per manufacturer’s protocol (ADCC reporter assay, Promega). Target cells were generated by transiently transfecting HEK293 suspension culture with the full-length WT-spike construct including the transmembrane domain but lacking the C-terminal 19 amino acids which contains an endoplasmic reticulum (ER)-retention signal that had been found to reduce incorporation into pseudovirus [34]. Cells were harvested after 72 h and seeded at 25,000 cells/well and at 1:3 ratio with reporter cells and purified IgG incubated at indicated concentrations. Luminescence was measured after 6 h incubation.

### Antibody Dependent Enhancement (ADE) assay

Spike-bearing viral pseudoparticles were produced through co-transfection with the above full length WT-spike construct along with the lentiviral plasmids pMDLg/pRRE, pRSV-REV (a kind gift from Dr Wang-Cheng-I, Singapore Immunology Network) and the luciferase reporter plasmid pHIV-Luc [35] into HEK293 adherent cells and harvested after 4 days. 25 μl of pseudovirus-bearing supernatant was mixed with antibody at indicated concentrations and Raji, THP-1 or ACE2 expressing CHO-cells at 25,000 cells/well and incubated at 37°C in a CO_2_ incubator. Media was changed after 24 h and luminescence expression measured after a further 24 h by washing the cells in PBS and adding reagent (Luciferase Assay System, Promega)

### Efficacy testing for SARS-CoV-2 infection in K18-ACE2 mice

All animal work was monitored by and performed in accordance with the protocol approved by the DSO Institutional Animal Care and Use Committee (IACUC) and the Institutional Biosafety Committee (IBC). B6.Cg-Tg (K18-ACE2)2Prlmn/J) were obtained from Jackson Laboratory (JAX). Mice for the studies were female between 7 and 12 weeks old. To determine the day of peak viral load, following acclimatization of the mice to the isocages, groups of 3 mice were anesthetized individually with 3% isoflurane using the precision vaporiser and infected intranasally (I.N.) with 50 μl of 1.2×10^4^ TCID50 of SARS-CoV-2. Following infection, the mice were transferred to new isocages. On the indicated days three mice were euthanised by carbon dioxide asphyxiation. The lungs were harvested, weighed and made to 10% w/v with viral grow medium then mashed through a disposable mesh using a plunger. This mixture was aliquoted into screw cap tubes and stored at -80°C, for later determination of lung viral load by qualitative real-time PCR (qRT-PCR) and cell culture to determine the tissue culture infective dose (TCID), and cytokine/chemokine mRNA expression. To assess therapeutic efficacy, mice were anesthetised at the indicated time points with 3% isoflurane using a precision vaporiser and treated with indicated concentration of antibody in 200 μl PBS by intra-peritoneal (I.P.) injection, and mice were returned to the isocages for recovery. Lungs were harvested for determination of viral load and cytokine/chemokine mRNA expression levels at the peak virus day. For the survival groups, mice were weighed when indicated and returned to their isocage. Any mice that showed >20% weight loss or significant inactivity were euthanized humanly using carbon dioxide asphyxiation.

### Determination of lung viral load in infected mice

Lung viral load was determined using TCID_50_ in VERO E6 cells, or by qRT-PCR for detecting viral RNA (genome copy number; GCN). To determine lung viral load, serially-diluted lung homogenates were incubated with 2×10^4^ Vero E6 cells in total of 100 μl of culture media (MEM/2% FCS) in 96-well flat bottom plates and incubated for 5 days. Virus titre, reciprocal to cell viability, was measured using Viral Toxglo reagent (Promega) to determine cell viability relative to uninfected (cells only) controls. TCID_50_ was subsequently determined using Reed-Muench method. To determine the viral GCN, RNA was extracted from lung homogenates using QIAamp Viral RNA mini kit (Qiagen). Viral RNA was detected using primers and probes targeted against ORF1ab as previously described [36] with 7500 Fast Real-Time PCR system (Applied Biosystem). GCN was determined against standard controls included within each RT-PCR run.

### Cytokine and chemokine mRNA measurements

RNA was extracted from lung homogenates harvested at 3 dpi using QIAamp Viral RNA mini kit. cDNA was synthesized using High-Capacity cDNA reverse transcription kit (Thermo Fisher Scientific) with the addition of RNase inhibitor (RNaseOUT, Thermo Fisher Scientific). Cytokine and chemokine expression was determined using TaqMan Fast Universal PCR mastermix (Thermo Fisher Scientific) with PrimeTime® Standard qPCR assays (Integrated DNA Technologies) for CCL2 (Mm.PT.58.42151692), CXCL10 (Mm.PT.58.43575827), IL1b (Mm.PT.58.41616450), IL6 (Mm.PT.58.10005566), TNF (Mm.PT.58.12575861), IFNγ1 (Mm.PT.58.30132453.g) and normalized using GADPH (Mm.PT.39a.1) levels. Fold change was determined using the 2^-δδCt^ method comparing anti-SARS-CoV2 specific or isotype control monoclonal antibodies-treated / irrelevant isotype treated mice, to uninfected mice controls.

## Cytokine and chemokine protein measurements

Cytokine and chemokine protein levels in mouse serum were determined by ELISA using paired antibodies for mouse IFNγ (Invitrogen, Cat# 88–7314), IL2 (Invitrogen, Cat# 88–7024), IL6 (Invitrogen, Cat# 88–7064) and CCL2 (R&D Systems, Cat# DY479-05), according to the manufacturer’s instructions. Briefly, 384 well plates were coated with 1X capture antibody for 16 h at 4°C. After blocking for 1 h with blocking buffer provided in the kits, mouse serum was added to the plate and incubated for 2 h at room temperature. Plates were washed thrice with washing buffer and incubated for 1 h with detection antibody, followed by 1 h incubation with Streptavidin-HRP. After washing, plates were developed with colorimetric detection substrate 3,3′,5,5′-tetramethylbenzidine (Turbo-TMB; Pierce). The reaction was stopped with 2M sulphuric acid, and absorbance was measured at 450 nm.

### Efficacy testing in Golden Syrian hamsters

This study was performed in strict accordance with the recommendations in the Guide for Care and Use of Laboratory Animals, Eighth Edition (National Academy Press, Washington, D.C., 2011). The University of Texas Medical Branch (UTMB) facility where this study was conducted is accredited by the Association for Assessment and Accreditation of Laboratory Animal Care. The protocol was approved by the UTMB Institutional Animal Care and Use Committee (Protocol Number: 2005062) and complied with the Animal Welfare Act, the U.S. Public Health Service Policy, and other Federal statutes and regulations related to animals and experiments involving animals. All efforts were made to minimize suffering. Biosafety Level 3 tasks were performed by appropriately trained personnel in the Galveston National Laboratory. Golden Syrian hamsters (n = 12, 7–8 weeks old, equal sex) were obtained from Envigo. Animals were group housed (three per cage) in micro-isolator cages for the duration of the study (Days 0–7). Standard rodent chow and bedding were routinely supplied, changed, and monitored. All hands-on manipulations, including dose administration and biosampling, were performed while animals were under inhalation isoflurane sedation (1–5%). Prior to study, hamsters were implanted subcutaneously with IPTT-300 programmable transponders for identification and temperature monitoring.

On Day 0, hamsters were administered 5×10^5^ TCID_50_ SARS-CoV-2 (USA_WA1/2020) via intranasal instillation (50 μl per nare). The virus suspension was prepared on the day of challenge from frozen seed stock initially generated (one passage) in Vero C1008 (E6) cells (BEI Resources, NR-596, Lot 3956593) from lyophilized material provided by the World Reference Center for Emerging Viruses and Arboviruses at UTMB (TVP 23156). Next generation sequencing confirmed 100% consensus sequence-level match to the original patient specimen (GenBank accession MN985325.1). At 4 hpi, hamsters were administered sterile saline (vehicle control, n = 6) or SC31 (n = 6) in sterile saline (20 mg/kg) via intraperitoneal injection. Animals were monitored and scored once daily for clinical signs of disease including alterations in appearance/posture, body weight, subcutaneous body temperature, and activity/behavior. Prospectively defined criteria that required immediate euthanasia included dyspnea and/or cyanosis, reluctance to move when stimulated, and/or 20% weight loss. Nasal cavity samples, collected daily using 0.5 mm Microbrush^®^ Applicators, were placed into 0.1 ml sterile phosphate buffered saline for viral load analysis. At scheduled study termination seven days post-challenge or upon meeting endpoint criteria, animals were humanely euthanized via carbon dioxide inhalation followed by bilateral thoracotomy. From each hamster, lungs were removed, weighed, and processed for viral load and histopathology. For viral load analysis, half of the left lung lobe was excised, homogenized, and clarified via centrifugation. Clarified homogenate was analyzed for infectious virus and viral RNA via TCID_50_ and qRT-PCR assays as described below. For histopathologic analysis, remaining lung tissue was placed into 10% neutral buffered formalin for 24 h after which formalin was replaced and samples were allowed to fix for at least another 21 days. Fixed tissues were processed to hematoxylin and eosin-stained slides and examined by a board-certified pathologist at Experimental Pathology Laboratories, Inc. (EPL®) in Sterling, Virginia. Findings were graded from minimal to severe based on a standardized scoring scale (S3 Table in S1 File).

### Efficacy testing in Indian rhesus macaques

This study was performed in strict accordance with the recommendations in the Guide for Care and Use of Laboratory Animals, Eighth Edition (National Academy Press, Washington, D.C., 2011). The University of Texas Medical Branch (UTMB) facility where this study was conducted is accredited by the Association for Assessment and Accreditation of Laboratory Animal Care. The protocol was approved by the UTMB Institutional Animal Care and Use Committee (Protocol Number: 2005062) and complied with the Animal Welfare Act, the U.S. Public Health Service Policy, and other Federal statutes and regulations related to animals and experiments involving animals. All efforts were made to minimize suffering. Biosafety Level 3 tasks were performed by appropriately trained personnel in the Galveston National Laboratory. Indian rhesus macaques (*Macaca mulatta*, 23–36 months old, equal sex) were obtained from Envigo. Prior to receipt, animals were surgically implanted with vascular access ports (VAP) by the vendor. VAP maintenance, including weekly flushing, was performed by UTMB veterinary staff. Animals were individually housed in stainless steel nonhuman primate caging equipped with automatic water and squeeze backs for the duration of the study. Certified primate Diet 5048 was provided to the animals daily. To promote and enhance the psychological well-being of the macaques, food enrichment consisting of fresh fruits and vegetables was provided daily. Environmental enrichment including various manipulatives (Kong toys, mirrors, and puzzles) was also provided. Animals were randomized into the four study groups (n = 6 per group; equal sex) using body weight as a stratification factor. All hands-on manipulations, including dose administration and biosampling, were performed while animals were sedation via ketamine (5 mg/kg)/dexmedetomidine (0.025 mg/kg) intramuscular injection.

On Day 0, macaques were administered 1×10^6^ TCID_50_ SARS-CoV-2 (USA_WA1/2020) via combined mucosal atomization (1 mL as delivered using a MAD Nasal^TM^ Intranasal Mucosal Atomization Device per manufacturer instructions) and intratracheal instillation (4 mL). Intratracheal instillations were performed as described previously [37]. The virus suspension was prepared on the day of challenge from frozen seed stock initially generated (one passage) in Vero C1008 (E6) cells (BEI Resources, NR-596, Lot 3956593) from lyophilized material provided by the World Reference Center for Emerging Viruses and Arboviruses at UTMB (TVP 23156). Next generation sequencing confirmed 100% consensus sequence-level match to the original patient specimen (GenBank accession MN985325.1). Four hours post-infection, macaques were administered sterile phosphate buffered saline (PBS; vehicle control, n = 6) or SC31 in sterile PBS (5, 10, or 20 mg/kg; n = 6 per group) through the implanted VAP. Animals were monitored and scored twice daily for clinical signs of disease including alterations in activity/appearance (i.e., hunched posture), food consumption/waste output, and outward changes in breathing patterns. Prospectively defined criteria that required immediate euthanasia included severe dyspnea and/or agonal breathing and prostate posture/reluctance to move when stimulated. No animals met endpoint criteria during study. Nasal cavity samples, collected using sterile cotton-tipped medical swabs, were placed into 0.5 mL sterile PBS for viral load analysis. For bronchoalveolar (BAL) fluid collection, animals were sedated as previously described and placed in ventral recumbency. The trachea was visualized and cannulated by an appropriately sized rubber feeding tube. Following the placement of the feeding tube, 20 ml of sterile PBS was introduced into the lung and recovered. This was repeated for a total of 40 ml per animal. Total collected volumes ranged from 10–30 ml. Collected BAL fluid was centrifuged under ambient conditions (10 min at 500 g) after which the supernatant was removed. The resulting cell pellet was resuspended in sterile 1 ml sterile PBS for viral load analysis. At scheduled study termination timepoints (two, six, or 10 days post-challenge), select animals from each group were humanely euthanized via intravenous administration of a pentobarbital-based euthanasia solution under deep anesthesia followed by bilateral thoracotomy. From each macaque, representative samples of upper and lower lung, bronchial lymph nodes, trachea, kidney, and spleen tissue were collected, homogenized, clarified via centrifugation, and analyzed for infectious virus and viral RNA via TCID_50_ and qRT-PCR assays as described below.

### Viral load determination in samples collected during hamster and macaque studies

Samples collected during hamster and macaque studies were analyzed for viral load via TCID_50_ and qRT-PCR assays. For TCID_50_ analysis, samples were serially diluted and incubated with 2×10^4^ Vero C1008 (E6) cells (BEI Resources, NR-596, Lot 3956593) cells in 100 μl of culture medium (MEM/2% FBS) in 96-well flat bottom plates (4–5 replicate wells per dilution). Each plate contained negative and positive control wells inoculated with culture medium and diluted virus stock, respectively. Cultures were incubated at 37°C/5% CO_2_ for 96h after which cytopathic effect was measured via microscopic observation. The TCID_50_ for each sample was calculated as previously described [38]. For qRT-PCR analysis, samples (50 μl) were added to TRIzol® LS Reagent (250 μl) and allowed to incubate under ambient conditions for 10 min. Samples were processed to RNA using Zymo Direct-zol^TM^ RNA Mini Prep kits per manufacturer instructions. RNA samples were analyzed via qRT-PCR targeting the SARS-CoV-2 E gene. Primers were ordered from Invitrogen and probe was ordered from Integrated DNA Technologies. Probe was labeled as the 5’-end with fluorophore 9-carboxyfluroescein (6-FAM) and included an internal quencher (ZEN) and a 3’-end quencher (IowaBlackFQ, IABkFQ). Master Mix was prepared by combining forward primer (250 nM, 5’-ACAGGTACGTTAATAGTTAATAGCGT-3’), reverse primer (250 nM, 5’-ATATTGCAGCAGTACGCACACA-3’), and probe (375 nM, 5’-6FAM-ACACTAGCC/ZEN/ATCCTTACTGCGCTTCG-IABkFQ-3’) with 12.5 μL of 2X QuantiFast Probe Mix (QIAGEN), 0.25 μL of 2X QuantiFast RT Mix (QIAGEN), and PCR-grade water (fill to 20 μL). To the Master Mix, test sample (5 μL) was added resulting in a final volume of 25 μL per reaction. Real-time analysis was performed using the Bio-Rad CFX96^TM^ Real-Time PCR Detection System. Thermocycling conditions were as follows: Step 1, 1 cycle, 50°C for 10 minutes; Step 2, 1 cycle, 95°C for 10 minutes; Steps 3–5, 45 cycles, 95°C for 10 seconds, 60°C for 30 seconds, single read. For quantification purposes, viral RNA extracted from the virus seed stock was used.

## Results

### SC31, a potent neutralizing antibody isolated from a convalescent patient, binds to a conserved region of RBD of the SARS-CoV-2 SP

Anti-SARS-CoV-2 antibodies were generated by single B cell antibody interrogation of PBMCs from convalescent COVID-19 patients. SP-binding IgG^+^ B cells were sorted directly from patient PBMC samples obtained at 15 and 27 days post symptom onset and cultured to induce antibody secretion. Despite sampling soon after the onset of symptoms, SP-specific IgG B cells were detected at a frequency below 1% of total IgG^+^ B cells (S1A Fig). An analysis of antibody binding to wild-type SP ectodomain in the B cell culture supernatants identified a total of 36 SP binding antibodies, of which ten had significant neutralizing activity (S1B Fig). The heavy and light chain antibody pairs from six of these clones could be isolated and were converted to full IgG1 antibodies (S1 Table in S1 File).

**Fig 1:**
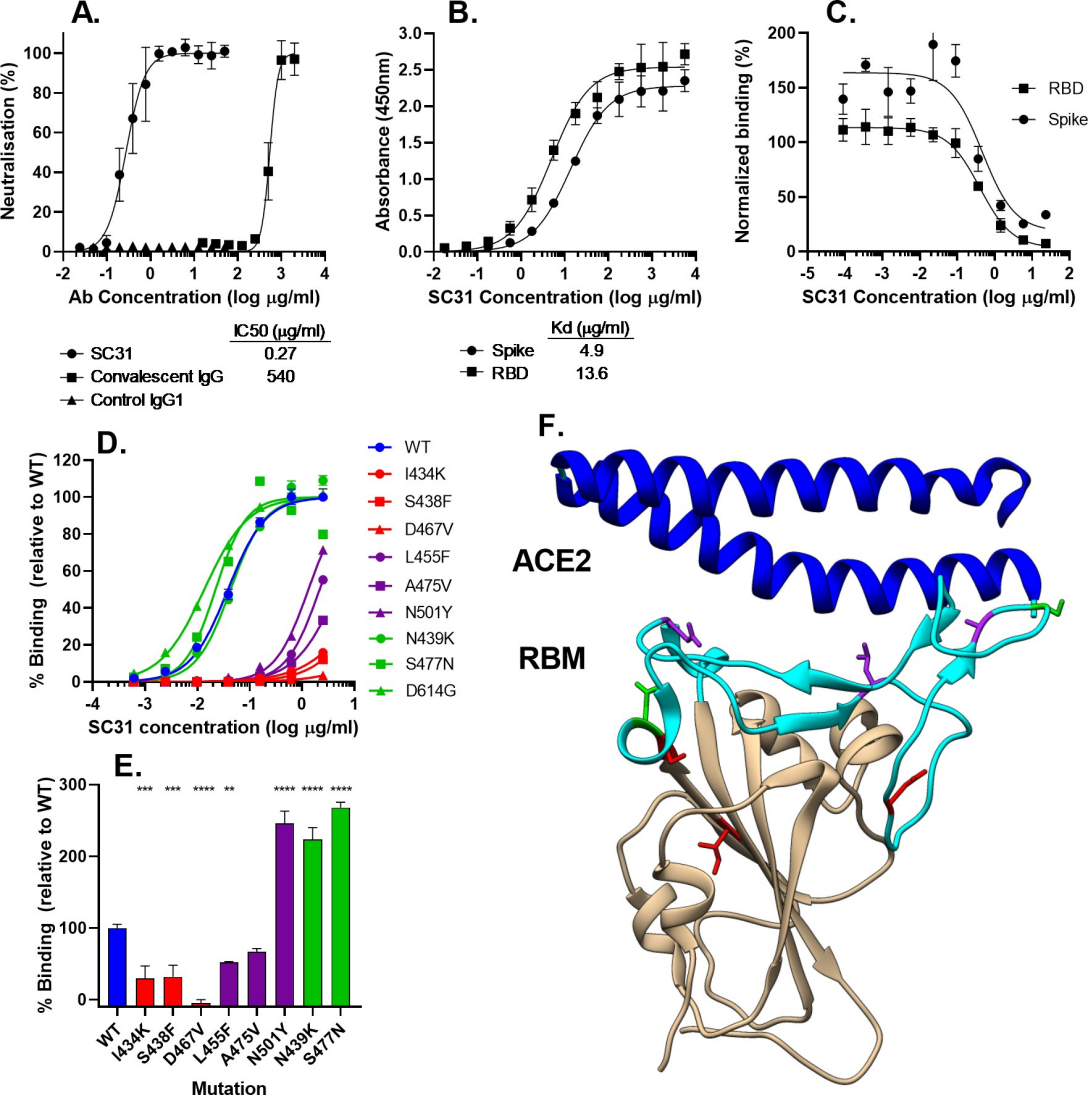
SC31 binds SARS-CoV-2 SP and neutralizes virus through inhibition of binding to ACE2. (**A**) Neutralization of 100 TCID_50_ of infectious virus by SC31 IgG1 compared to control IgG1 or IgG purified from donor serum, represented as a percentage relative to uninfected and “virus only” controls. (**B**) Binding affinity of SC31 IgG to purified wild type SP or RBD as measured by ELISA. (**C**) Inhibition of wild-type SP or RBD binding to cells expressing membrane-bound ACE2 by SC31 IgG1 at different concentrations as measured by flow cytometry, expressed as a percentage relative to “no antibody” and “no viral protein” controls. (**D**) Binding affinity of SC31 as determined by ELISA to purified wild-type SP (blue) and SP mutants that either do not affect SC31 or ACE2 binding (green), affect SC31 but not ACE2 binding (purple) or affect both SC31 and ACE2 binding (red). (**E**) Binding affinity of purified wild-type and mutant SP to hACE2-expressing CHO cells based on fluorescence intensity measured by flow cytometry. Statistical significance was determined versus wild-type SP for each mutant by one-way ANOVA: ** p<0.01, *** p<0.001, **** p<0.0001. (**F**). Locations of single amino acid mutations (green/purple/red) on a crystal structure of RBD showing the interaction of the RBM (cyan) with ACE2 N-terminal helix (blue). All results represent the mean of three independent replicates with bars showing standard error.

SC31, isolated from the patient at Day 27 post symptom onset, was the most potent antibody in terms of SARS-CoV-2 neutralization in Vero E6 cell culture with an IC_50_ of 0.27μg/ml (1.85nM) (S1C Fig). Significantly, a comparison to IgG purified from the plasma fraction of its corresponding early convalescent patient sample showed that SC31 was 2000-fold more potent (Fig 1A). SC31 bound to the ectodomain and RBD of SARS-CoV-2 SP with similar affinity (Fig 1B), indicating that an inhibition of receptor binding is likely, in part, the mechanism of neutralization. Indeed, SC31 demonstrated concentration-dependent inhibition of the interaction of both ectodomain and RBD of SARS-CoV-2 SP with human ACE2 (Fig 1C).

To further explore its mechanism of action, the structure-function of the epitope recognized by SC31 was investigated by evaluating the binding of SC31 to naturally occurring SP mutants. A total of 36 single amino acid SP mutations identified from publicly available databases were tested [17, 18]. We focused on 23 mutations that were either within the receptor binding motif (RBM, aa438-506) and predicted to make direct contact with ACE2 receptor, or were beyond the RBM and existed at high frequencies. The majority of these mutations were found to have minimal impact on the binding affinity of SC31 to SP and the ACE2 receptor (S2 Fig). Significantly, the two most common circulating mutations within the RBM, N439K and S477N, as well as the D614G mutation (which delineates a major viral clade and is by far the most frequent SP mutation) did not cause any significant changes in its affinity (Fig 1D). Six mutations (I434K, S438F, D467V, L455F, A475V, N501Y) that resulted in significant loss of SC31 binding were identified. Three of these mutations, I434K, S438F and D467V, also resulted in significant or total loss of SP binding to the ACE2 receptor (Fig 1E). These suggest that the observed loss of antibody binding was due to a destabilization of the entire RBM structure. Importantly, due to reduced fitness, the virus strains with these mutations would be unlikely to propagate efficiently. The other three mutations minimally reduced or, in the case of N501Y, enhanced ACE2 receptor binding and would be expected to lead to viral escape due to an alteration of the binding epitope. The locations of these three mutations are highlighted on a recently published crystal structure [39] and are likely to represent part of the SC31 binding epitope (Fig 1F). Critically, none of these three SP mutants that appeared early in the pandemic appear to be commonly circulating, perhaps due to other negative effects on the protein stability. Thus, we conclude that SC31 binds to a stable epitope in the RBD of the SARS-CoV-2 SP that is well-conserved in commonly circulating SARS-CoV-2 variants.

### SC31 requires Fc-mediated effector functions for maximal therapeutic efficacy against SARS-CoV-2 infection and does not cause ADE in K18-hACE2 mice

As with other IgG1 antibodies, the binding of the SC31 to FcγR on immune cells is likely to stimulate beneficial Fc-mediated effector functions including antibody-dependent cellular cytotoxicity (ADCC), antibody-dependent cell phagocytosis (ADCP), antibody-dependent cell-mediated virus inhibition (ADCVI) and complement-dependent cytotoxicity (CDC). However, these have the potential to cause an ADE of infection and disease. The risk of ADE is especially a concern when therapeutic antibodies reach sub-neutralizing concentrations. To ameliorate the risk of ADE, the use of an Fc isotype that does not bind to FcγRs can be explored. However, equally important is an investigation of how an abrogation of Fc-mediated functions might affect the therapeutic efficacy of an anti-SARS-CoV-2 antibody.

**Fig 2:**
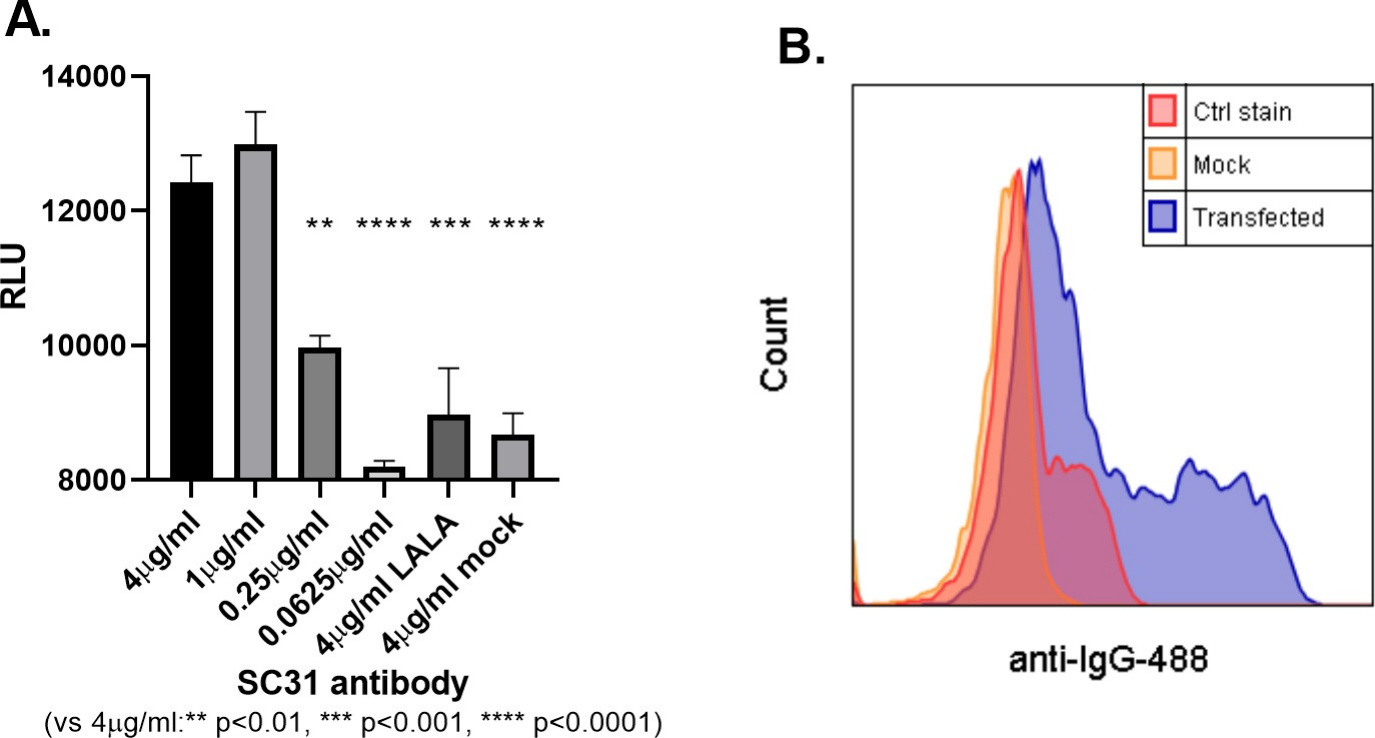
SC31 induces ADCC. (**A**) Activation of ADCC signaling by SC31 or Fc null-binding LALA variant at various concentrations incubated with FcγRIIIa-expressing reporter cell line and SP- or mock-transfected HEK293, as determined by luciferase expression. Statistical significance in comparison to the highest SC31 concentration of 4 μg/ml was determined by one-way ANOVA. (**B**) Specific binding of SC31 to SP-transfected HEK293 cells in comparison to mock-transfected cells or fluorophore stain only (Ctrl stain).

To determine the role of Fc-mediated effector functions in the therapeutic efficacy of SC31, the abilities of SC31 and its FcγR null-binding (LALA) variant to induce Fc effector-mediated activity were compared. The upstream activation of the FcγRIIIa ADCC signalling pathway was evaluated using a Jurkat reporter cell line [40] after co-culture with target HEK293 cells expressing membrane-bound SARS-CoV-2 SP. In contrast to its LALA variant, SC31 was confirmed to induce a dose-dependent activation of ADCC signalling (Fig 2).

Subsequently, the therapeutic efficacies of SC31 and its LALA variant were evaluated in K18-hACE2 mice to determine the contribution of Fc-mediated functions to the antibody’s therapeutic efficacy. Severe disease manifestations following infection with SARS-CoV-2 infection have been demonstrated in K18-hACE2 transgenic mice [20, 41–43]. Mice infected intranasally with 1.2 x 10^4^ TCID_50_ (nCoV-19/Singapore/3/2020) presented with severe disease manifestations including lethargy, weight loss, overexpression of proinflammatory cytokines/chemokines (IL-6, CXCL10, and CCL2) and, ultimately, death between 6 and 8 days post infection (dpi) with associated high virus titers in lung tissue (S3 Fig).

**Fig 3:**
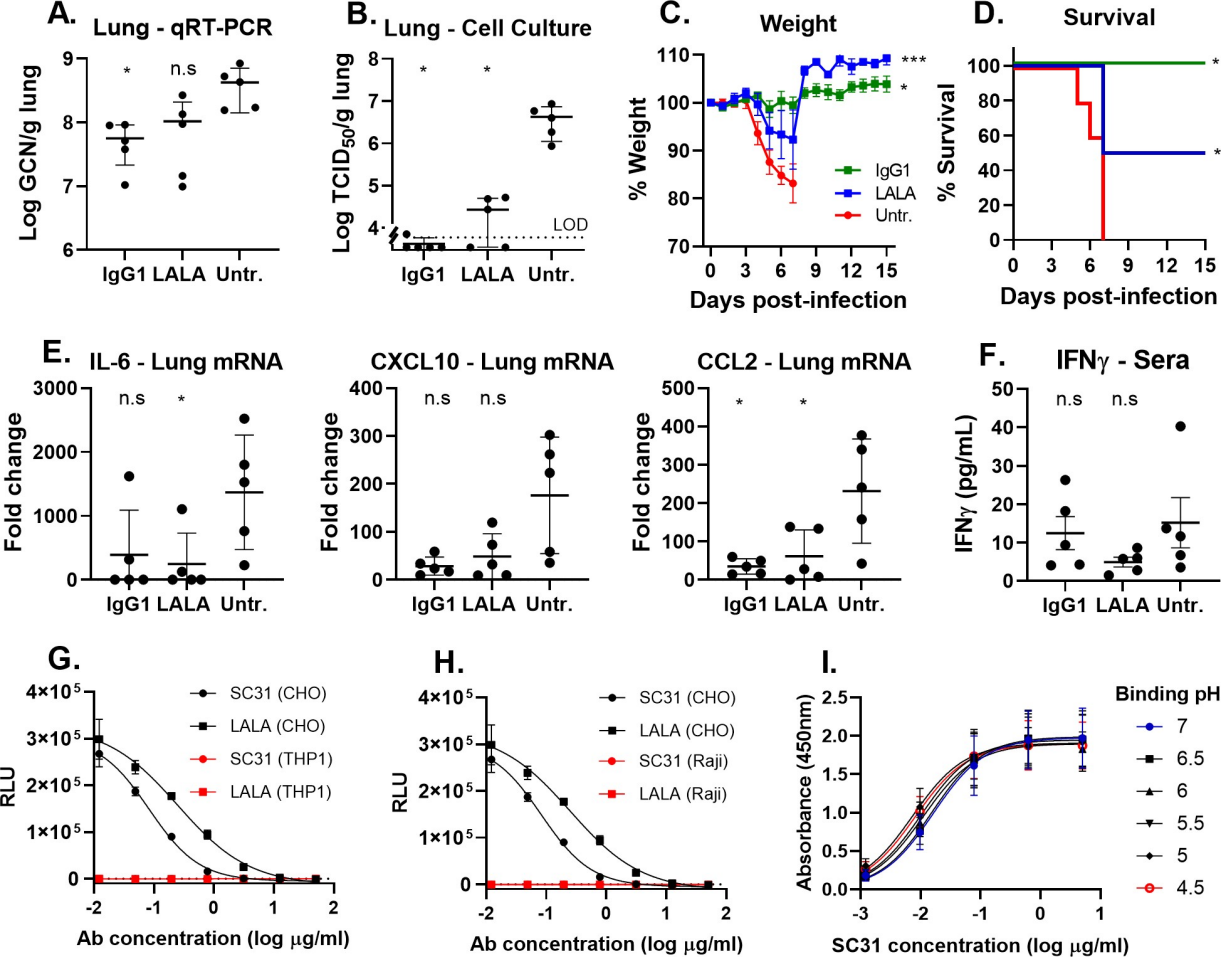
SC31 requires Fc effector functions for optimal therapeutic benefit. SARS-CoV-2 infected K18-hACE2 mice, 10 mice per group, were dosed with either 20 mg/kg SC31 IgG1 or LALA variant antibodies at 6 hpi. Half of mice (n = 5) were sacrificed at 3 dpi to ascertain lung viral load and cytokine levels in addition to serum antibody titers; the remaining mice were monitored for weight changes and survival. Lung viral load in IgG1-, LALA-treated or untreated mice at 3 dpi as measured by (**A**) qRT-PCR or (**B**) cell culture, the limit of detection (LOD) is indicated by the dotted line. Disease progression in infected mice as indicated by (**C**) weight loss and (**D**) survival. (**E**) Lung cytokine mRNA expression determined by qRT-PCR represented as fold-change over uninfected mice. (**F**) Sera IFNγ protein levels determined by sandwich ELISA. Each point represents one mouse and all error bars show standard error. (**G** and **H**) Lack of ADE of SARS-CoV-2 pseudovirus infection based on luciferase reporter gene expression following co-incubation with SC31 or LALA variant in (**G**) THP-1 and (**H**) Raji as compared to ACE2-expressing CHO cells. (**I**) Retention of SC31 binding affinity for wild-type SP between pH 4.5–7.0 as measured by ELISA. Results represent the mean of three or four independent replicates with error bars showing standard error. Statistical significance was determined using two-way ANOVA with Fisher’s LSD test for viral load, D5-7 weight loss or cytokines/chemokines and Chi-Square for D15 survival percentage, ns–p>0.05, * p<0.05, ***<0.001 vs. untreated.

SC31 or its LALA variant (20 mg/kg) were administered via intraperitoneal (IP) injection 6 hours post virus infection (hpi) to groups of 10 mice. At 3 dpi, lungs from half of the mice per group were harvested for viral load quantification and cytokine/chemokine mRNA and protein expression analyses. The remaining mice were monitored for survival and weight loss for a further 15 days. Quantification of viral RNA and infectious virus showed that the both LALA and IgG1 variants reduced viral load in a statistically significant manner compared to untreated (Fig 3A and 3B), which correlated with decreased weight loss and improved survival (Fig 3C and 3D), thus demonstrating a clear therapeutic benefit with either variant of SC31. Comparison between the LALA-treated and either IgG1-treated or untreated groups showed a significant difference in the day 15 endpoint survival rates and body weight across D5-7 but not for viral load. Nevertheless, there was a clear trend in viral load with the mean of the LALA group intermediate between IgG1-treated and untreated. Taken together, the results indicate that Fc-functionality enhances therapeutic efficacy above that provided by neutralization alone.

We also observed a similar though not statistically significant trend from cytokine/chemokine analysis of virus-infected mice with the LALA-treated group showing intermediate levels of CXCL10 and CCL2 between IgG1-treated and untreated (Fig 3E). IL-6 levels were reduced in both IgG1 and the LALA-treated groups following virus challenge. Intriguingly, the protein levels of IFNγ were markedly increased in mice treated with wild-type SC31 antibody (Fig 3F), suggesting that although the innate pro-inflammatory response was decreased, there was likely a beneficial targeted antiviral response driven by Fc-receptor engagement. Taken together, these results indicate that the abilities of SC31 to inhibit the binding of SARS-CoV-2 to ACE2 and induce a Fc-mediated IFN-γ response are both essential for its optimal therapeutic efficacy.

To investigate the risk of ADE, SC31 and its LALA variant were tested *in vitro* at sub-neutralizing concentrations using SARS-CoV-2 pseudovirus and THP-1 and Raji cell lines. ADE has been previously been modelled for SARS-CoV pseudovirus in these same cell lines [27, 44]. Importantly, no pseudovirus infection was observed in both THP-1 and Raji cells for either antibody at all the concentrations tested. This indicates that, despite its potent Fc-mediated effector functions, SC31 is unlikely to mediate ADE (Fig 3G and 3H). It has been suggested that the pH-selective binding of antiviral antibodies (i.e. lower binding affinity to the viral target at lower pH levels) may predict a risk of ADE as antibodies might dissociate from the virus in the low pH environment of endosomes. This loss of antibody binding to the virus could then allow viral entry into the cell. We found that SC31 maintains a high affinity for SP even at pH levels as low as 4.5 (Fig 3I), which might explain the lack of ADE of this potently neutralizing antibody.

### SC31 provides potent dose- and time-dependent therapeutic efficacy against SARS-CoV-2 infection in K18-hACE2 mice

**Fig 4:**
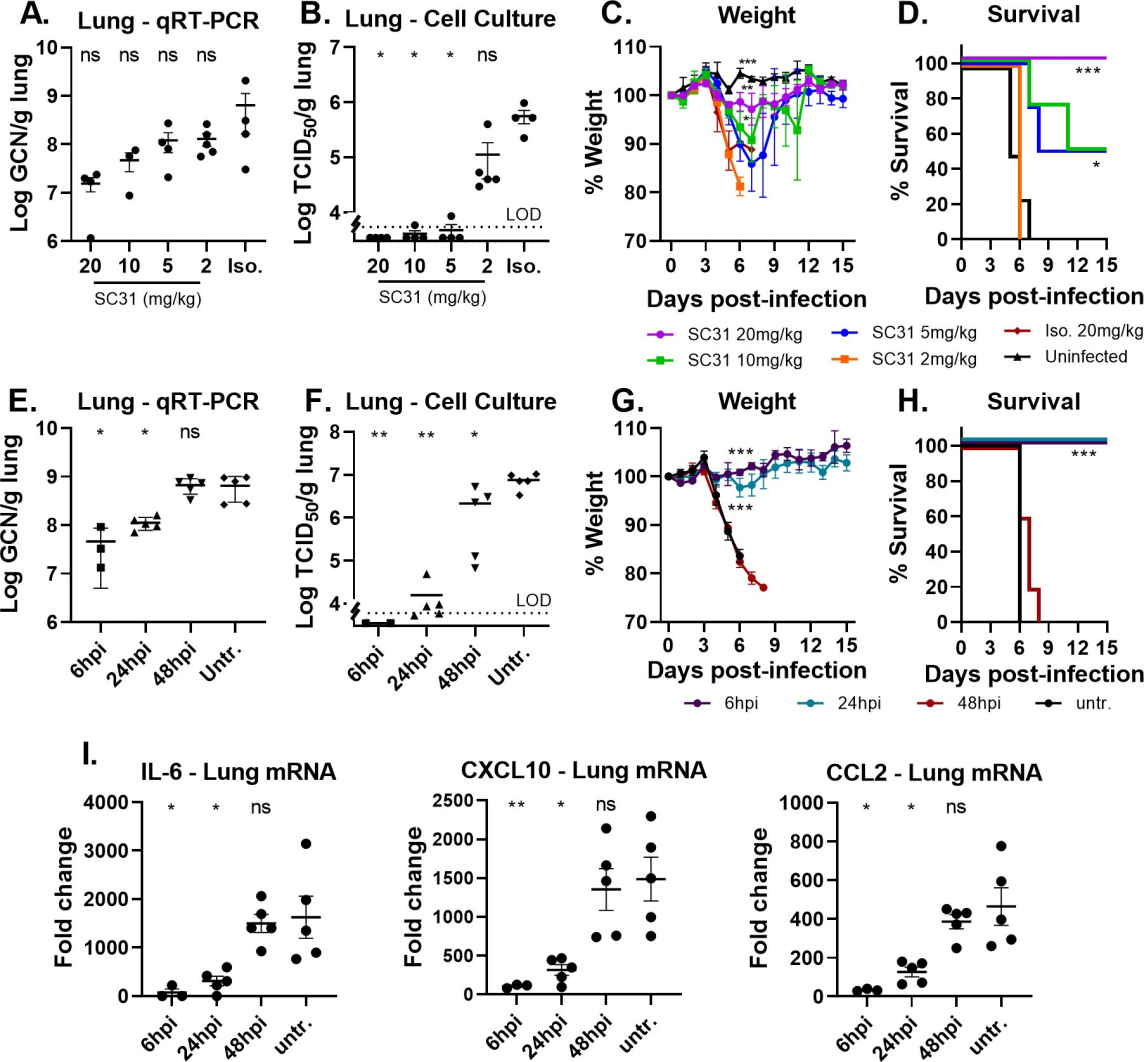
Dose-and time-dependent therapeutic benefit of SC31. Groups of 10 SARS-Cov-2 infected K18-hACE2 mice were treated with SC31 IgG1 or isotype control at the indicated doses 6 hpi. Half of the mice (n = 5) were sacrificed at 3 dpi to assess lung viral load; the remaining mice were monitored for weight changes and survival. Lung viral load as measured by (**A**) qRT-PCR and (**B**) cell culture, the limit of detection (LOD) is indicated by the dotted line. Disease progression is indicated by (**C**) weight loss and (**D)** survival. For the time-dependent study, SC31 IgG1 was administered at 20 mg/kg at the indicated time points. Half of the mice (n = 5) were sacrificed at 3 dpi to assess lung viral load and cytokine levels in addition to serum antibody titers. Remaining mice were monitored for weight changes and survival. Lung viral load as measured by (**E**) qRT-PCR and (**F**) cell culture, the limit of detection (LOD) is indicated by the dotted line. Disease progression in infected mice as indicated by (**G**) weight loss and (**H**) survival. (**I**) Lung cytokine mRNA expression determined by qRT-PCR and represented as fold-change over uninfected mice. Each point represents one individual mouse and all error bars show standard error. Statistical significance was determined using two-way ANOVA with Fisher’s LSD test for viral load, D5-6 weight loss or cytokines/chemokines and Chi-Square for D15 survival percentage, ns–p>0.05, * p<0.05, **p<0.01, ***p<0.001 vs. untreated.

The elucidation of the minimum effective dose and optimal timing for the therapeutic treatment of SARS-CoV-2-specific antibodies remains critical towards the successful establishment of a therapeutic strategy in the clinical setting. To further evaluate the therapeutic potency and dose response of SC31, K18-hACE2 mice were treated with 2, 5, 10, or 20 mg/kg at 6 hpi. At 3 dpi, half the mice were sacrificed for the quantification of lung viral RNA, infectious virus, and cytokine/chemokine expression. The survival and weight of the remaining mice were monitored until 15 dpi when the study was terminated. The level of viral RNA in lung tissues showed between 0.8–1.5-log reduction in a dose-dependent manner (Fig 4A). A corresponding reduction in infectious virus level was observed with a greater than three log reduction with 5 mg/kg antibody, and down to the limit of detection for the majority of mice treated with 20 mg/kg (Fig 4B). A dose-dependent reduction in weight loss, starting at 3 dpi, was observed in all virus-infected mice (Fig 4C). A similar dose-dependent effect on survival was observed (Fig 4D), with no mortality in animals treated with the highest dose (20 mg/kg), 50% mortality in mice dosed with either 5 or 10 mg/kg, and no survival at the lowest dose (2 mg/kg). All surviving mice overcame clinical signs of disease by 13 dpi as evidenced by the return to their pre-infection weight. Taken together, these results suggest that SC31 has therapeutic benefit at doses above 5mg/kg.

To ascertain the efficacious dosing window of SC31, infected K18-hACE2 mice were treated with 20 mg/kg of SC31 at 6, 24 and 48 hpi. As before, half of the mice were sacrificed for the quantification of lung viral RNA, infectious virus, and cytokine/chemokine expression. The well-being of the remaining mice was monitored until 15 dpi. All mice treated at 6 and 24 hpi survived with minimal weight loss while mice treated at 48 hpi lost weight and succumbed to disease at the same rate as untreated mice (Fig 4G and 4H). Lung viral RNA, infectious virus, and cytokine/chemokine (IL-6, CXCL10 and CCL2) levels followed the same trend with similar therapeutic benefit observed in mice treated at 6 and 24 hpi, while results in mice treated at 48 hpi were similar to those in untreated mice (Fig 4E, 4F and 4I). Taken together, results indicate that the efficacious dosing window in this model is before 48 hpi, *i*.*e*., prior to the peak of viral infection and inflammation that was observed at Day 3 (S3 Fig). Early clinical dosing, before the onset on lung inflammation, may thus be necessary for optimal therapeutic benefit.

### SC31 provides potent therapeutic benefit, protecting against severe COVID-19-like disease in Golden Syrian hamsters infected with SARS-CoV-2

To confirm the efficacy of SC31 in another disease model, against a second SARS-CoV-2 virus strain, and investigate the ability of SC31 to inhibit progression to severe lung inflammation, the Golden Syrian hamster model was used. This model shows severe disease manifestations with SARS-CoV-2 infection, including predictable weight loss, alveolar inflammation and bronchiolo-alveolar hyperplasia [45, 46]; and has been used for the evaluation of other COVID-19 antibody treatment candidates [17, 47].

**Fig 5:**
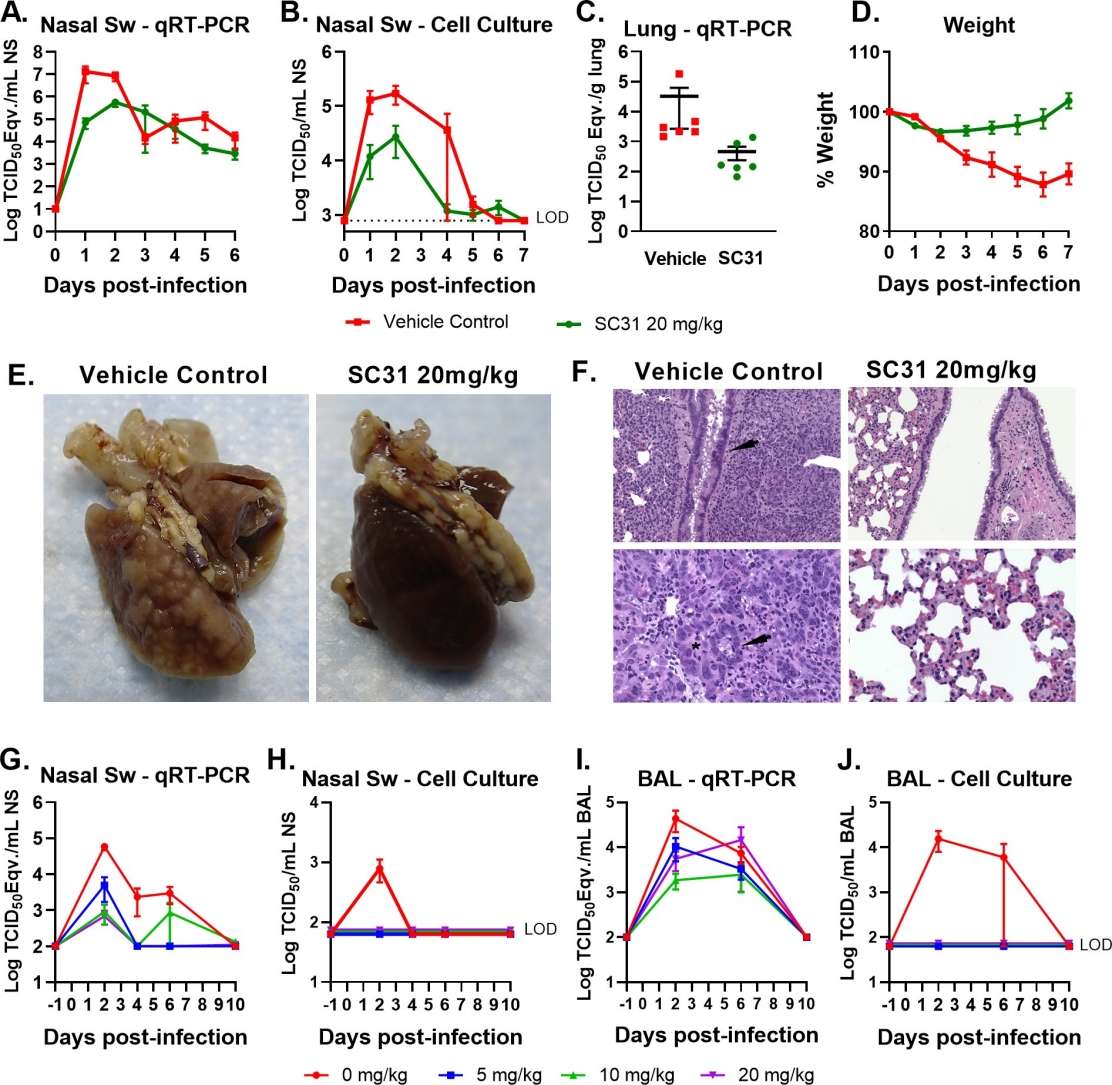
SC31 minimizes disease after SARS-CoV-2 challenge in Golden Syrian hamsters and Indian Rhesus Macaques. Twelve hamsters (6 male/6 female) were intranasally challenged with 5×10^5^ TCID_50_ SARS-CoV-2; 6 animals were treated with SC31 antibody or vehicle at 4 hpi. The viral load of nasal swabs (Nasal Sw) collected daily was determined by (**A**) qRT-PCR and (**B**) cell culture; dotted line represents the lower limit of detection (LOD) for the assay. (**C**) Viral load in hamster lungs harvested 7 dpi as determined by qRT-PCR. (**D**) Disease progression, as indicated by weight loss, was prevalent in vehicle controls. (**E)** Harvested lungs from vehicle control-treated (left) and SC31-treated (right) hamsters at 7 dpi; images taken following removal of the left lobe for viral load analysis; representative images are shown. (**F**) Histopathology examination of fixed lung tissue from vehicle control-treated (left) and SC31-treated (right) hamster at 7 dpi; representative images are shown. Indian rhesus macaques (12 male/12 female) were challenged with 1×10^6^ TCID_50_ SARS-CoV-2 via combined intratracheal/intranasal (mucosal atomization) delivery. Viral loads in Nasal Sw (**G-H**) and BAL fluid samples (**I-J**) collected at early, mid, and late stages of disease were determined by qRT-PCR and cell culture. For all panels, error bars show standard error.

To evaluate the therapeutic efficacy of SC31, hamsters (6 male/6 female) were intranasally challenged with 5 x 10^5^ TCID_50_ SARS-CoV-2 (USA_WA1/2020) followed by treatment with SC31 (20 mg/kg) or vehicle control (six hamsters per group). Body weights were monitored and nasal swabs were collected daily for SARS-CoV-2 RNA, and infectious virus analyses before the harvest of lungs at 6–7 dpi. When analyzed for total and infectious viral loads, nasal swabs from SC31-treated hamsters showed a 1–2 log_10_ reduction when compared to that of vehicle-treated hamsters (Fig 5A and 5B). SC31-treated hamsters showed a minor drop in weight that returned to pre-infection weight by Day 6 (Fig 5D). In contrast, vehicle-treated hamsters continually lost weight through Day 6, losing an average of ~10% of their pre-infection weight. Lungs collected from the vehicle-treated control hamsters at 6 to 7 dpi consistently showed higher total viral loads than those derived from SC31-treated hamsters (Fig 5C). In addition, average lung weights from vehicle-treated controls were higher, potentially indicative of virus-associated pulmonary edema (S2 Table in S1 File). Vehicle-treated control hamsters also presented with mottled, dark red lungs with 1–3 mm lesions diffusely distributed throughout the tissue, which correlate histologically to hemorrhage and bronchiolo-alveolar hyperplasia, respectively (Fig 5E). In comparison, only one of the six SC31-treated hamsters had this gross lung phenotype.

Additional histopathological findings in the vehicle control-treated group include an increase in type II pneumocytes indicative of acute lung damage, hyperplastic and hypertrophied bronchial/bronchiole epithelium (Fig 5F, top left, arrow) and alveoli lined by type II pneumocytes (bottom left, arrow) and enlarged nuclei (bottom left, asterisk). Conversely, microscopic findings associated with bronchial/bronchiole epithelium (Fig 5F, top right) and alveolar epithelium (Fig 5F, bottom right) were normal or less severe in SC31-treated hamsters. These findings were supported through histopathology severity grading of sections (S3 Table in S1 File) from each individual animal in the vehicle control treated (S4 Table in S1 File) and SC31-treated (S5 Table in S1 File) groups. Results from this study demonstrate that SC31 can effectively treat acute viral infection and limit the development of severe lung inflammation in the Golden Syrian hamster model.

### SC31 provides therapeutic benefit and eliminates infectious virus in a rhesus macaque model of COVID-19

The rhesus macaque (*Macaca mulatta*) has been shown to recapitulate COVID-19 in humans with respect to viral replication in the upper and lower respiratory tracts, and lung pathology [48–52]. While disease is mild when compared to smaller models such as mice and hamsters, rhesus macaques are more closely related to humans and, as such, have been extensively used to study COVID-19 medical countermeasures [53–56]. To evaluate the therapeutic efficacy of SC31 in this model, Indian rhesus macaques (12 male/12 female) were challenged with 1×10^6^ TCID_50_ SARS-CoV-2 (USA_WA1/2020) via combined intratracheal instillation and mucosal (intranasal) atomization delivery followed by intravenous administration of clinical grade antibody (5, 10, or 20 mg/kg) or sterile PBS at 4 hpi (six macaques per group). Animals were monitored daily for clinical signs of disease including changes in activity/behavior, appetite, and respiratory function. Nasal swabs and bronchoalveolar lavage (BAL) fluid were collected from all animals prior to virus challenge (baseline) and at 2, 4 (nasal swabs only), 6, and 10 dpi representing early, mid, and late stages of disease in the model. At 2, 6, and 10 dpi, two macaques per group were humanely euthanized following sample collection for tissue harvest and processing.

Total viral loads as measured via qRT-PCR in nasal swabs and BAL fluid were 1–2 log_10_ lower (dose-dependent) in SC31-treated animals versus macaques administered sterile PBS (Fig 5G and 5I). In a similar fashion, total viral RNA loads in collected tissues were higher in animals treated with PBS alone (S4 Fig). Infectious viral loads in the same samples collected from SC31-treated macaques were undetectable whereas samples from PBS-treated animals reached upwards of 4 log_10_ TCID_50_/mL during the early and midpoint stages of disease (Fig 5H and 5J). Infectious viral loads in tissue homogenates were below quantifiable levels in most samples, although low levels (< 500 TCID_50_/mL of homogenate) were present in upper lung tissue collected from animals in the PBS- and SC31 (5 mg/kg)-treated groups during the acute phase of disease (data not shown). These results demonstrate that SC31 decreases total viral burden and eliminates infectious virus in the upper and lower respiratory tracts following SARS-CoV-2 challenge in rhesus macaques.

## Discussion

There is an urgent need for the development of safe and efficacious therapeutic antibodies to address the global COVID-19 pandemic. Such antibodies represent a potentially critical treatment option in the absence of a widely available vaccine, especially for elderly and other at-risk groups. While some monoclonal antibodies have been granted Emergency Use Authorization, their mechanism(s) of action and the possibility of ADE at sub-therapeutic levels are still unclear.

In this study, we present the generation and characterization of a highly efficacious anti-SARS-CoV-2 IgG1 neutralizing antibody, SC31. Significantly, we show that the therapeutic efficacy of SC31 is not solely due to its ability to neutralize the virus but also involves Fc-mediated effector functions. The binding site of SC31 was mapped to the RBD of the SP and was shown to be conserved across all common circulating SARS-CoV-2 mutants, including the most common variant, D614G. The role of Fc-mediated effector activity on the therapeutic efficacy of the antibody was evaluated together with its potential to cause ADE. SC31 showed no evidence of being able to mediate ADE.

Although IgG1 isotype antibodies are predicted to bind stimulatory FcγR and trigger FcγR signaling as well as Fc-mediated effector functions (e.g., ADCC, ADCVI and CDC), not all IgG1 anti-SP antibodies exhibit the same capacity to elicit Fc-mediated effector functions. It has been shown that some anti-SP IgG1 antibodies exhibit weak or even negligible ADCC activity [16, 57]. This may explain, at least in part, the variation between *in vitro* and *in vivo* potencies reported for different anti-SP antibodies as well as the lack of immunity afforded by higher anti-SP IgG titers in some COVID-19 patients [29]. Importantly, this also suggests that in addition to neutralizing activities, the ability of an antibody to promote Fc-mediated effector functions and signaling may also be a key factor in therapeutic efficacy. Critically, SC31 was clearly able to trigger Fc-mediated effector functions, as evidenced by activation of ADCC signaling in contrast to an Fc-effector null LALA variant, without evidence of ADE at sub-therapeutic levels.

This ability of SC31 to induce Fc-mediated effector signaling was subsequently demonstrated to be essential for the optimal therapeutic efficacy of the antibody. When SC31 and its LALA variant were evaluated in an *in vivo* mouse model of SARS-CoV-2 infection, the LALA variant was observed to have much reduced therapeutic efficacy–with only 50% compared with 100% survival for SC31 LALA and WT variants, respectively. This correlated with higher lung viral load as determined by total and infectious virus analyses, suggesting that the LALA variant was not able to clear virus, thereby resulting in greater inflammatory responses. This indicates that FcγR engagement of lung phagocytic cells by SC31 did not result in excessive pro-inflammatory signaling, but also appeared to induce a more targeted and robust antiviral response characterized by higher IFNγ levels. The reduction in systemic pro-inflammatory markers including IL-6, CCL2 and CXCL10 is likely due to a reduction in viral load that dampens the systemic inflammatory signaling through various innate viral pathogen recognition receptors such as Toll-Like Receptor 7 [58]. The increase in IFNγ levels, however, may reflect the engagement of a targeted NK and T-cell driven antiviral response, as these are the primary producers of IFNγ. There may also be other Fc-mediated pathways such as CDC which contribute to therapeutic efficacy that could be explored in future studies.

These benefits were also observed in the hamster model where treatment with SC31 IgG1 resulted in significantly reduced lung pathology and a higher peak temperature as compared to controls, suggesting a more robust antiviral response. In summary, although a recent study showed that anti-SP IgG from COVID-19 patients with severe disease can promote hyperinflammatory responses in alveolar macrophages [59], our data indicate that the situation is likely more complicated and depends on multiple parameters that contribute to the overall function of virus-induced antibodies. In the case of SC31, such parameters are unlikely to pose a risk of systemic immune dysregulation when used as a therapeutic.

Severe disease and mortality in COVID-19 appears to be driven by excessive inflammation due to a failure of the immune system to control viral infection in the lungs [60]. SC31 showed potent dose-dependent therapeutic efficacy above 5 mg/kg. However, once the inflammatory cascade is triggered, SC31 was no longer able to exert a therapeutic effect as evidenced by the poor outcome when mice were treated at 48hrs which is close to the peak of cytokine response at Day 3. This indicates that antibody therapy is best administered prior to the onset of severe symptoms.

Overall, we have demonstrated that the anti-SP IgG1 antibody SC31, generated from an early convalescent patient 27 days after symptom onset, is able to control infection in three animal models of COVID-19 disease by decreasing/eliminating viral load and protecting against lung damage, and has the potential to be a highly efficacious therapeutic in the clinical setting. This efficacy is driven by the dual mechanisms of potent neutralization of SARS-CoV-2 infection through blocking SP binding to the human ACE2 receptor and induction of a robust antiviral response driven by Fc-mediated effector functions, but importantly, without concomitant ADE. While much remains to be understood regarding the mechanism of action(s) of antibodies in immune responses against SARS-CoV-2, it is clear that SC31 has great potential as a COVID-19 therapeutic antibody and is now undergoing clinical trials.

## Supporting information

S1 Fig Discovery of SC31 from single B cells. (**A**) Dot plot showing gating strategy for sorting spike-binding IgG B cells from patient PBMCs samples HJ57 and CSY63. (**B**) Neutralization of 25 TCID_50_ of live SARS-CoV-2 coronavirus by single B cell culture supernatants showing spike-binding activity. Each supernatant was tested in duplicate and is shown individually. (**C**) Neutralization efficacy of single cell IgG1 antibodies against 100 TCID_50_ live SARS-CoV-2 coronavirus represented as a percentage respective to uninfected and virus infected cell controls. EC50 values for antibodies showing significant neutralization are shown. Results represent the mean of four independent replicates with bars indicating standard error.
(TIF)

S2 Fig Determination of SC31 binding to spike variants. (**A**) Binding affinity of SC31 to purified wild-type spike and spike mutants as determined by ELISA. Results are the mean of three independent replicates and are represented as a percentage of maximal absorbance against wild-type spike at the highest antibody concentration. (**B**) Binding affinity of purified wild-type and mutant spike protein to hACE2-expressing CHO cells as determined by fluorescence intensity with flow cytometry. Results are the mean of three independent replicates with bars showing the standard error and are represented relative to wild-type spike binding to ACE2. Only mutations within the RBD region were tested.
(TIF)

S3 Fig Establishment of K-18 human ACE2 transgenic mouse SARS-CoV-2 infection model. (**A**) Disease progression in K18 mice as shown by weight loss (left) and survival (right). (**B**) Kinetics of viral infection in K18 mice with lung viral load based on genome copies (left) and infectious disease (right). The dotted line indicates the limit of detection (LOD). (**C**). Kinetics of the cytokine response in the lung as measured by mRNA expression of pro-inflammatory cytokines IFNβ, TNF, IL1b, IL6 and chemokines CCL2, CXCL10 represented as fold-change over uninfected mice. Each point represents one individual mouse with the mean indicated by the horizontal lines or bars. Statistical significance between viral load on adjacent days was determined using Student’s t-test.
(TIF)

S4 Fig SC31 reduces viral load in tissues following SARS-CoV-2 challenge in Indian Rhesus Macaques. Following SARS-CoV-2 challenge, select tissues collected at scheduled necropsy were processed and analyzed for viral load via qRT-PCR. In general, virus titers were low across all six tissues analyzed. Quantifiable virus levels were consistently measured in the trachea and bronchial lymph nodes particularly during the acute phase of disease. (**A**) trachea (**B**) bronchial lymph nodes (**C**) kidney (**D**) spleen (**E)** upper lung (**F**) lower lung. For all panels, the dashed line represents the lower limit of detection (LOD); dots represent individual animals.
(TIF)

S1 File (PDF)
