# Supplementary figures and images for: The Fc-mediated effector functions of a potent SARS-CoV-2 neutralizing antibody, SC31, isolated from an early convalescent COVID-19 patient, are essential for the optimal therapeutic efficacy of the antibody
Source: PLoS One. 2021 Jun 23;16(6):e0253487. doi: 10.1371/journal.pone.0253487 (PMC8221499; doi:10.1371/journal.pone.0253487)

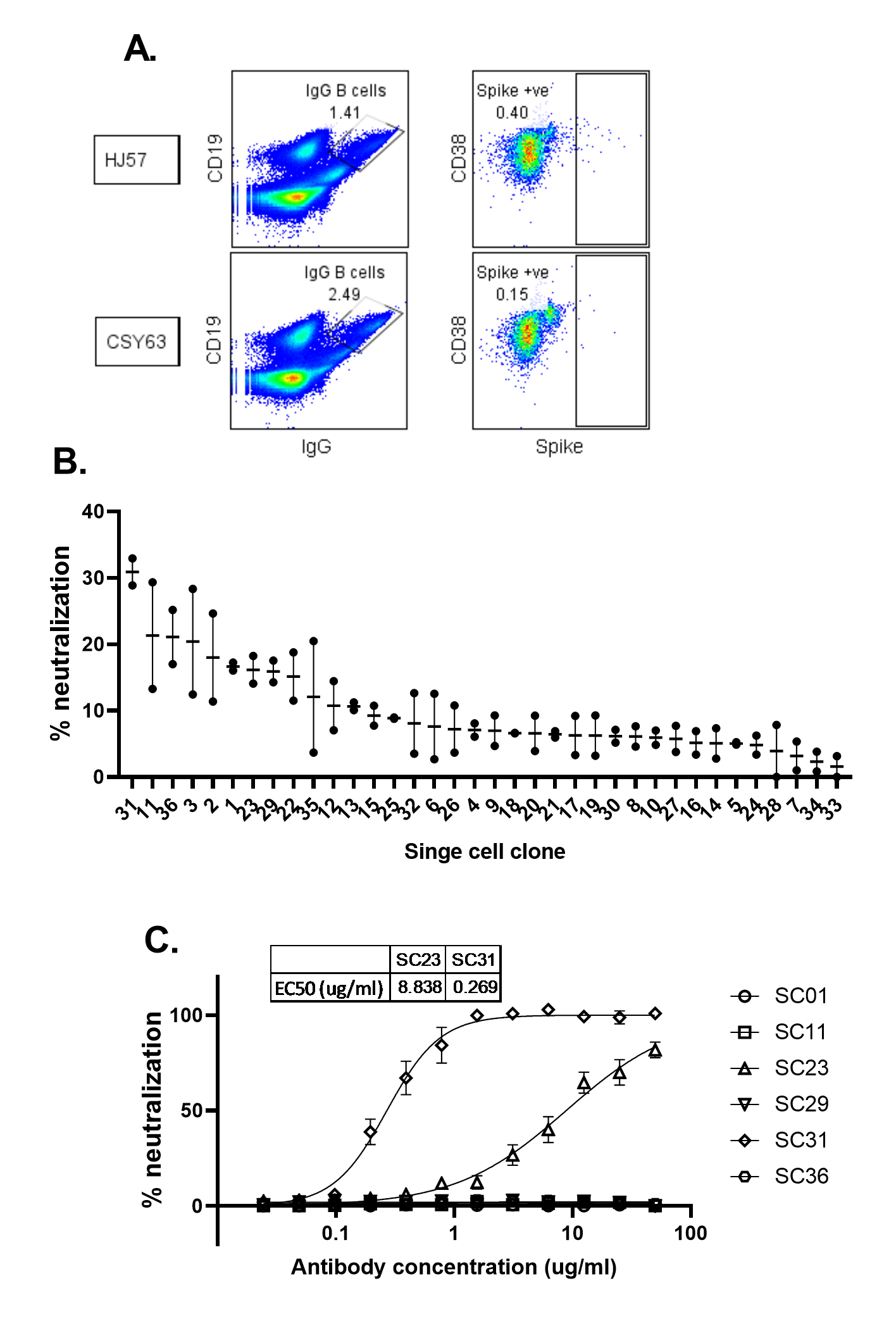

Supplement: S1 Fig — (A) Dot plot showing gating strategy for sorting spike-binding IgG B cells from patient PBMCs samples HJ57 and CSY63. (B) Neutralization of 25 TCID50 of live SARS-CoV-2 coronavirus by single B cell culture supernatants showing spike-binding activity. Each supernatant was tested in duplicate and is shown individually. (C) Neutralization efficacy of single cell IgG1 antibodies against 100 TCID50 live SARS-CoV-2 coronavirus represented as a percentage respective to uninfected and virus infected cell controls. EC50 values for antibodies showing significant neutralization are shown. Results represent the mean of four independent replicates with bars indicating standard error. (TIF) [file pone.0253487.s001.tif]

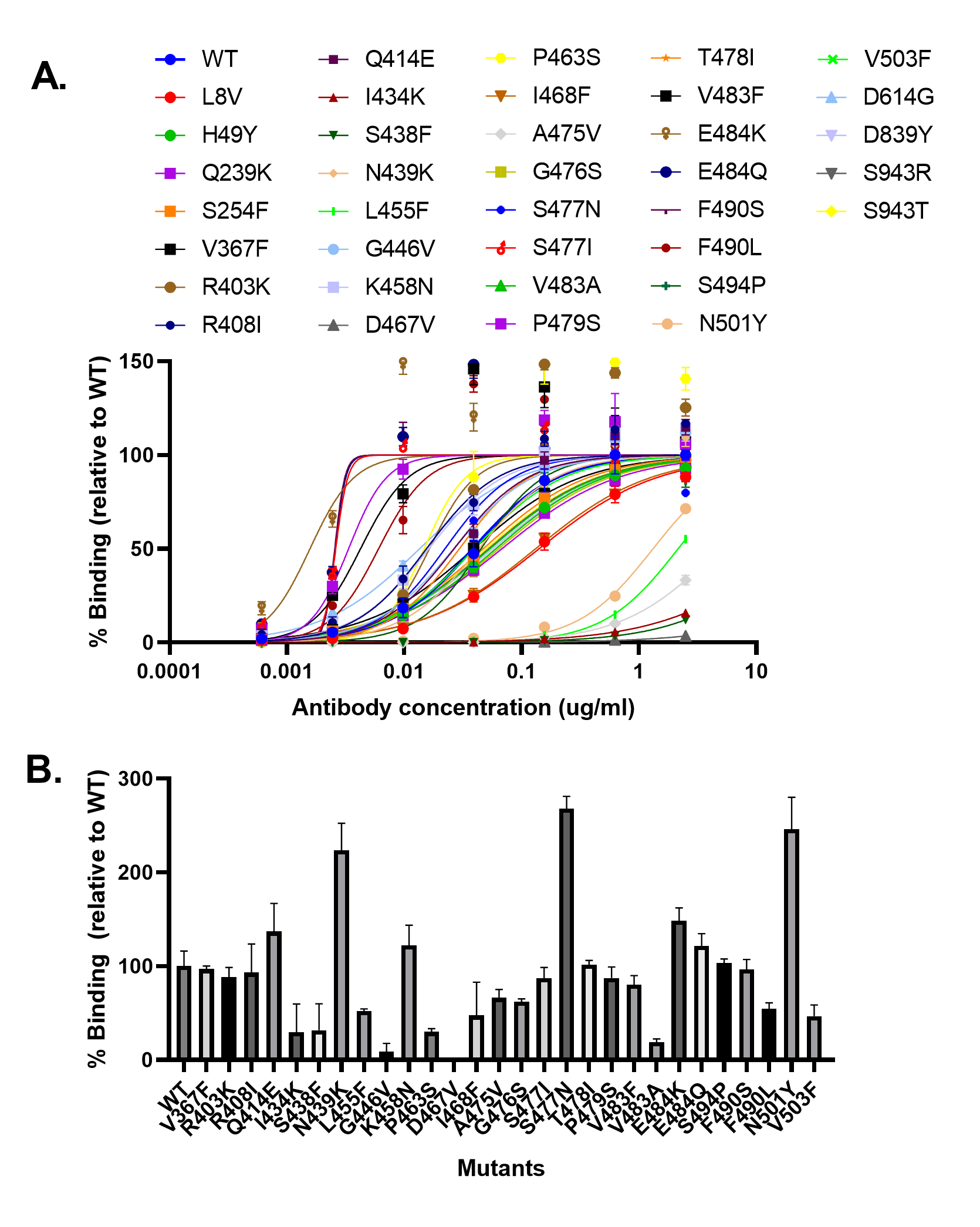

Supplement: S2 Fig — (A) Binding affinity of SC31 to purified wild-type spike and spike mutants as determined by ELISA. Results are the mean of three independent replicates and are represented as a percentage of maximal absorbance against wild-type spike at the highest antibody concentration. (B) Binding affinity of purified wild-type and mutant spike protein to hACE2-expressing CHO cells as determined by fluorescence intensity with flow cytometry. Results are the mean of three independent replicates with bars showing the standard error and are represented relative to wild-type spike binding to ACE2. Only mutations within the RBD region were tested. (TIF) [file pone.0253487.s002.tif]

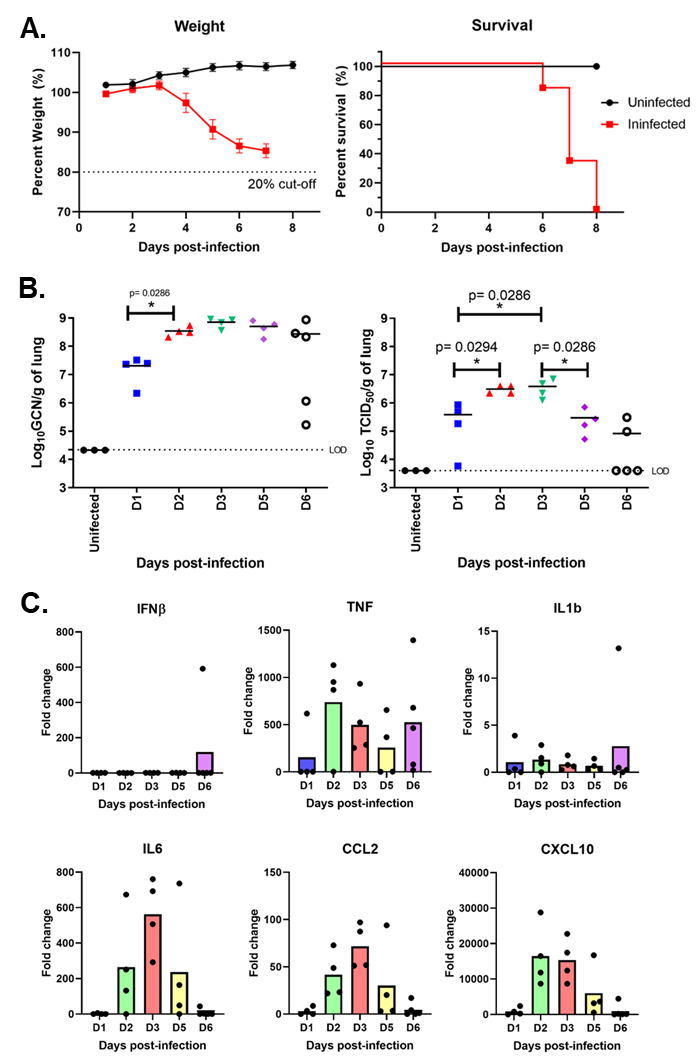

Supplement: S3 Fig — (A) Disease progression in K18 mice as shown by weight loss (left) and survival (right). (B) Kinetics of viral infection in K18 mice with lung viral load based on genome copies (left) and infectious disease (right). The dotted line indicates the limit of detection (LOD). (C). Kinetics of the cytokine response in the lung as measured by mRNA expression of pro-inflammatory cytokines IFNβ, TNF, IL1b, IL6 and chemokines CCL2, CXCL10 represented as fold-change over uninfected mice. Each point represents one individual mouse with the mean indicated by the horizontal lines or bars. Statistical significance between viral load on adjacent days was determined using Student’s t-test. (TIF) [file pone.0253487.s003.tif]

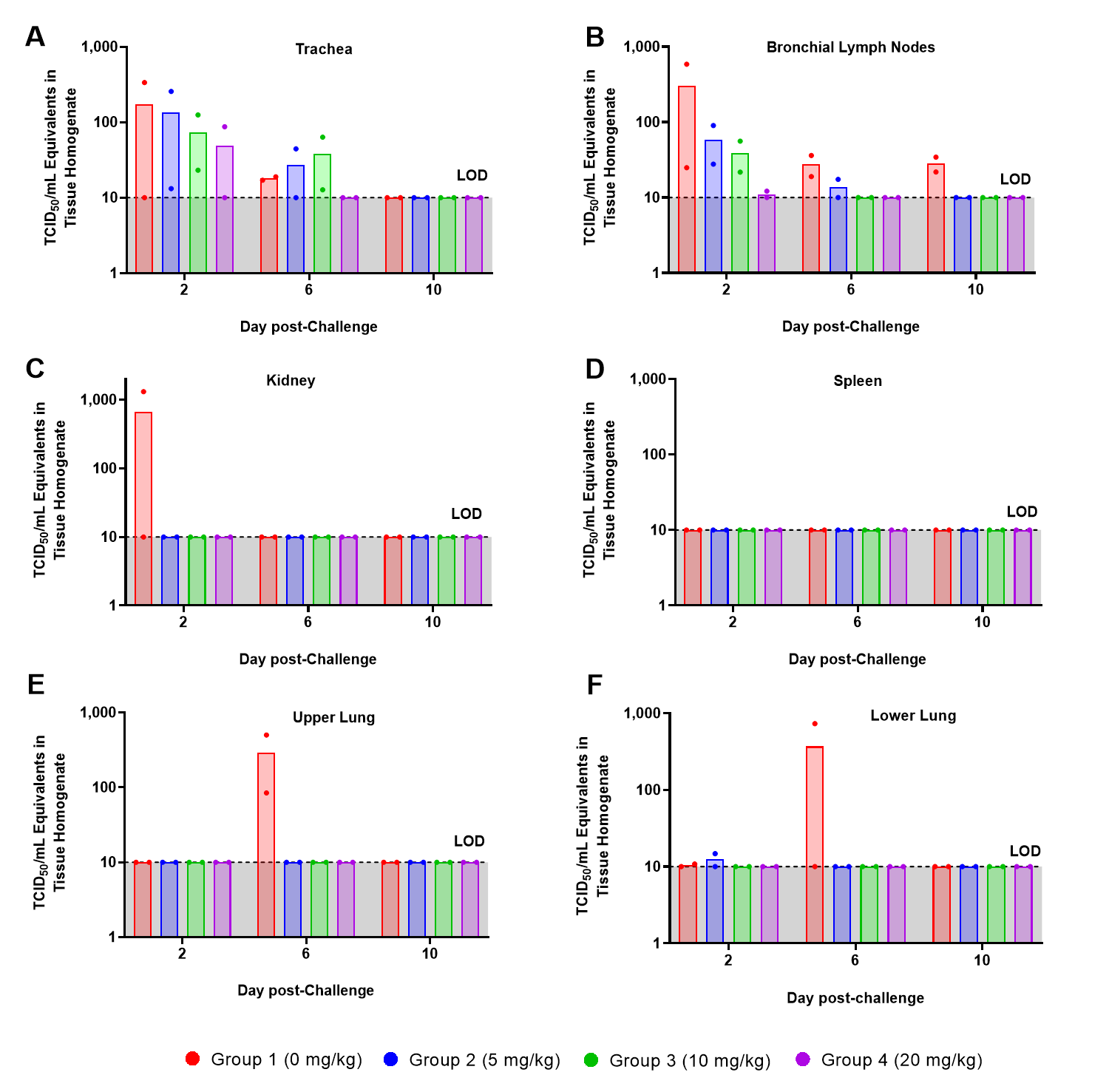

Supplement: S4 Fig — Following SARS-CoV-2 challenge, select tissues collected at scheduled necropsy were processed and analyzed for viral load via qRT-PCR. In general, virus titers were low across all six tissues analyzed. Quantifiable virus levels were consistently measured in the trachea and bronchial lymph nodes particularly during the acute phase of disease. (A) trachea (B) bronchial lymph nodes (C) kidney (D) spleen (E) upper lung (F) lower lung. For all panels, the dashed line represents the lower limit of detection (LOD); dots represent individual animals. (TIF) [file pone.0253487.s004.tif]
