# Supplementary material for: The Fc-mediated effector functions of a potent SARS-CoV-2 neutralizing antibody, SC31, isolated from an early convalescent COVID-19 patient, are essential for the optimal therapeutic efficacy of the antibody
Source: PLoS One. 2021 Jun 23;16(6):e0253487. doi: 10.1371/journal.pone.0253487 (PMC8221499; doi:10.1371/journal.pone.0253487)
Supplement: S1 File — (PDF) [file pone.0253487.s005.pdf]

Table S1: Amino acid sequence of the variable regions of the heavy and light chain pairs

Ab Chain	Amino Acid Sequence
SC01-VH	EVQLVQSGGGVVQPGRSLRLSCAASGFTFSSYGMHWVRQAPGKGLEWVAVISYDGSNKYYAD SVKGRFTISRDNSKNTLYLQMNSLRAEDTAVYYCAKDEEIVVVTATTVDVWGQGTTVTVSS
SC01-VL	ETALTQSPGTLSLSPGERATLSCRASQSVSSSYLAWYQQKPGQAPRLLIYGASSRATGIPDRFSGS GSGTDFTLTISRLEPEDFAVYYCQQYGSSPPSITFGQGTRLEIK
SC11-VH	EVQLVQSGPEVKKPGTSVKVSCKASGYTFSSYSMHWVRQAPGQSLEWMGWINGGSGNTKYSE KFQGRITISRDTSASTAYMELSRLRSDDTAVYYCARLYSGYDMWGQGTLVTVSS
SC11-VL	DVVMTQSPSSLSASVGDRVTITCRASQSISSYLNWYQQKPGKAPKLLIYAASSLQSGVPSRFSGS GSGTDFTLTISSLQPEDFATYYCQQSYSTPPNTFGQGTKLEIK
SC23-VH	EVQLVESGPTLVKPTQTLTLTCTFSGFSLSTSGVGVGWIRQPPGKALEWLALIYWDDDKRYSPS LKSRLTITKDTSKNQVVLTMTNMDPVDTATYYCAHRPRGYIYGAPFDYWGQGTLVTVSS
SC23-VL	QSVLTQPPSVSEAPRQRVTISCSGSSSNIGNNAVNWYQQLPGKAPKLLIYYDDLLPSGVSDRFSG SKSGTSASLAISGLQSEDEADYYCASWDDSLIGPVFGGGTKLTVL
SC29-VH	QVQLVQSGGGVVQPGRSLRLSCAASGFTFSYYGMHWVRQAPGKGLEWVAVISYDGSNKYYA DSVKGRFTISRDNSKNTLYLQMNSLRAEDTAVYYCARSDSGSYLPFDYWGQGTLVTVSS
SC29-VL	QSVVTQPASVSGSPGQSITISCTGTSSDVGGYNYVSWYQHHPGKAPKLMIYDVSNRPSGVSNRF SGSKSGNTASLTISGLQAEDEADYYCSSYTSSTTLAVFGGGTKLTVL
SC31-VH	QVQLVQSGGGLVQPGGSLRLSCAASGFTVSSNYMNWVRQSPGKGLEWVSVIYSGGSTFYADS VKGRFTISRDNSKNTLYLQMNSLRAEDTAVYYCARDLMEDGMDVWGQGTTVTVSS
SC31-VL	EIVLTQSPSSVSASVGDRVTITCRASQGISSWLAWYQQKPGKAPKLLIYVASSLQSGVPSRFSGS GSGTDFTLTISSLQPEDVATYYCQQANSFPLFGGGTKVEIK
SC36-VH	EVQLVQSGGGVVQPGRSLRLSCAASGFTFSNFGMHWVRQAPGKGLEWVAVISYDGSVQNYAD SVKGRFTISRDKSNNTLYLQMKSLRHEDTAVYFCVKGAQLDDYWGQGTLVTVSS
SC36-VL	DVVMTQSPLSLPVTLGQPASISCRSSQSLVYSDGNTYLSWFHQRPGQSPRRLIYKVSNRDSGVPD RFSGSGSGTDFTLKISRVEAEDVGVYYCMQTTHWPYTFGQGTKLEIK

Table S2: Total lung weights in vehicle control- and SC31-treated Golden Syrian Hamsters following SARS-CoV-2 challenge

Animal ID	Group	Day Post-Challenge[a]	Lung Weight[b]	Avg Lung Weight[c]
1	Vehicle Control	7	1.3	1.43 ± 0.23
2		7	1.3	
3		7	1.8	
4		7	1.6	
5		7	1.4	
6		6	1.2	
7	SC31-Treated	7	1.2	1.13 ± 0.18
8		7	1.1	
9		7	1.4	
10		7	1.0	
11		7	1.2	
12		7	0.9	

[a]Scheduled study termination was seven days post-challenge; animal 6 was euthanized six days post-challenge due to excessive weight loss

[b]In grams; standard deviation shown

[c]Standard deviation shown

Table S3: Lung histopathology grading scale

Grade	Descriptor	Description
1	Minimal	This corresponds to a histopathologic change ranging from inconspicuous to barely noticeable but so minor, small, or infrequent as to warrant no more than the least assignable grade. For multifocal or diffusely-distributed lesions, this grade was used for processes where less than approximately 10% of the tissue in an average high-power field was involved. For focal or diffuse hyperplastic/hypoplastic/atrophic lesions, this grade was used when the affected structure or tissue had undergone a less than approximately 10% increase or decrease in volume.
2	Mild	This corresponds to a histopathologic change that is a noticeable but not a prominent feature of the tissue. For multifocal or diffusely-distributed lesions, this grade was used for processes where between approximately 10% and 25% of the tissue in an average high-power field was involved. For focal or diffuse hyperplastic/hypoplastic/atrophic lesions, this grade was used when the affected structure or tissue had undergone between an approximately 10% to 25% increase or decrease in volume.
3	Moderate	This corresponds to a histopathologic change that is a prominent but not a dominant feature of the tissue. For multifocal or diffusely-distributed lesions, this grade was usd for processed where between approximately 25% and 50% of the tissue in an average high-power field was involved. For focal or diffuse hyperplastic/hypoplastic/atrophic lesions, this grade was used when the affected structure or tissue had undergone between an approximately 25% to 50% increase or decrease in volume.
4	Marked	This corresponds to a histopathologic change that is a dominant but not an overwhelming feature of the tissue. For multifocal or diffusely-distributed lesions, this grade was used for processed where between approximately 50% to 95% of the tissue in an average high-power field was involved. For focal or diffuse hyperplastic/hypoplastic/atrophic lesions, this was used when the affected structure or tissue had undergone between an approximately 50% to 95% increase or decrease in volume.
5	Severe	This corresponds to a histopathologic change that in an overwhelming feature of the tissue. For multifocal or diffusely-distributed lesions, this grade was used for processes where greater than approximately 95% of the tissue in an average high-power field was involved. For focal or diffuse hyperplastic/hypoplastic/atrophic lesions, this grade was used when the affected structure or tissue had undergone a greater than approximately 95% increase or decrase in volume.

Table S4: Lung histopathology in vehicle control-treated Golden Syrian Hamsters following SARS-CoV-2 challenge

**Lung Histopathology in Vehicle Control-Treated Golden Syrian Hamsters following SARS-CoV-2 Challenge**					
Animal ID	Group	Day Post-Challenge[a]	Histopathology Summary		
			Observation	Grade[b]	Right and/or Left Lung
1	Vehicle Control	7	Hemorrhage - alveolar	1	Right
			Hyperplasia - bronchiolo-alveolar	4	Both
			Inflammation, mixed cell - alveolar	2	Both
			Syncytial cell - present	n/a	Both
2	Vehicle Control	7	Hemorrhage - alveolar	1	Right
			Hyperplasia - bronchiolo-alveolar	3	Both
			Infiltrate, mononuclear cell - perivascular	1	Both
			Inflammation, mixed cell - alveolar	2	Both
			Syncytial cell - present	n/a	Both
3	Vehicle Control	7	Hemorrhage - alveolar	1	Both
			Hyperplasia - bronchiolo-alveolar	4	Both
			Hypertrophy	2	Both
			Inflammation, mixed cell - perivascular	2	Both
			Syncytial cell - present	n/a	Both
4	Vehicle Control	7	Hemorrhage - alveolar	2	Both
			Hyperplasia - bronchiolo-alveolar	5	Both
			Hypertrophy - mesothelium	2	Both
			Infiltrate, mononuclear cell - perivascular	1	Both
			Inflammation, mixed cell - bronchoalveolar	2	Both
			Minearlization	1	Right
			Syncytial cell - present	n/a	Both
5	Vehicle Control	7	Hemorrhage - alveolar	1	Both
			Hyperplasia - bronchiolo-alveolar	4	Both
			Hypertrophy - mesothelium	2	Both
			Infiltrate, mononuclear cell - perivascular	1	Both
			Inflammation, mixed cell - alveolar	2	Both
6	Vehicle Control	6	Hemorrhage - alveolar	1	Both
			Hyperplasia - bronchiolo-alveolar	4	Both
			Hypertrophy - mesothelium	2	Both
			Infiltrate, mononuclear cell - perivascular	1	Both
			Inflammation, mixed cell - alveolar	2	Both
			Syncytial cell - present	n/a	Both

[a]Scheduled study termination was seven days post-challenge; animal 6 was euthanized six days post-challenge based on weight loss criteria

[b]See Supplementary Table S2

Table S5: Lung histopathology in SC31-treated Golden Syrian Hamsters following SARS-CoV-2 challenge

Animal ID	Group	Day Post-Challenge[a]	Histopathology Summary		
			Observation	Grade[b]	Right and/or Left Lung
7	SC31-Treated	7	Hyperplasia - bronchiolo-alveolar	3	Both
			Infiltrate, mononuclear cell - perivascular	1	Both
			Inflammation, mixed cell - bronchoalveolar	2	Both
8	SC31-Treated	7	Hemorrhage - alveolar	2	Right
			Hyperplasia - bronchiolo-alveolar	3	Both
			Infiltrate, mononuclear cell - perivascular	1	Both
9	SC31-Treated	7	Hyperplasia - bronchiolo-alveolar	3	Both
			Infiltrate, mononuclear cell - perivascular	1	Both
			Inflammation, mixed cell - perivascular	2	Both
			Syncytial cell - present	n/a	Both
10	SC31-Treated	7	Hemorrhage (artifact)	2	Right
11	SC31-Treated	7	Hemorrhage - alveolar	1	Both
			Hyperplasia - bronchiolo-alveolar	4	Both
			Infiltrate, mononuclear cell - perivascular	1	Right
			Inflammation, mixed cell - alveolar	2	Both
			Syncytial cell - present	n/a	Both
12	SC31-Treated	7	No findings	n/a	n/a

[a]Scheduled study termination was seven days post-challenge

[b]See Supplementary Table S2
